# Biomechanical study of a new rim plate fixation strategy for two kinds of posterolateral depression patterns of tibial plateau fractures: a finite element analysis

**DOI:** 10.1186/s13018-023-04315-1

**Published:** 2023-11-07

**Authors:** Bin-bin Zhang, Bing-hao Wang, Jiong Mei, Cong-feng Luo, Yi Zhu

**Affiliations:** Department of Orthopaedic Surgery, Shanghai Sixth People’s Hospital Affiliated to Shanghai Jiao Tong University School of Medicine, 600 Yishan Road, Shanghai, 200233 China

**Keywords:** Tibial plateau fractures, Posterolateral depression fractures, Finite element analysis, Barrel hoop plate, Fracture modeling

## Abstract

**Purpose:**

The biomechanical capacity of “Barrel Hoop Plate (BHP)” in the treatment of the posterolateral tibial plateau (PL) depression fractures remains unknown. In this study, two kinds of posterolateral tibial plateau depression models involving mild slope-type depression fracture (MSDF) and local sink hole-type depression fracture (LSDF) were created to test and compare the biomechanical capacities of BHP with the other two conventional fixations (Anterolateral Plate and Posterolateral Plate, ALP and PLP) by finite element analysis.

**Methods:**

The 3D models of three kinds of plate-screw systems and the two kinds of PL-depression models (MSDF and LSDF) were created. An axial force of 400N was applied from the distal femur to the tibial plateau. The maximal displacements of the posterolateral fractures (PLFs), the distribution on the PLFs articular surface and key points displacements were measured. Stresses in the fixation complex including the maximal Equivalent (von-Mises) Stress of implants, the max shear stress of PLFs and stiffness of the fixation were calculated.

**Results:**

The maximal displacement of MSDF was least in Group BHP. The maximal displacement of LSDF was least in Group ALP. In MSDF, BHP showed the best rim fix effect in MSDF, but unsatisfactory results in LSDF. In both MSDF and LSDF, the greatest max Equivalent Stress of the plate and the screw occurred in the PLP system. ALP and BHP showed a comparable stiffness in MSDF and ALP had the strongest stiffness in the fixation of LSDF.

**Conclusions:**

In MSDF, the BHP has the best biomechanical capacity, especially in displacements of key points such as the PL rim, fracture line, and depression center. In LSDF, the ALP system shows the best biomechanical effect. Although the PLP has the best fixation effect on the posterior wall, it is not suitable for PL-depression fracture fixation.

**Supplementary Information:**

The online version contains supplementary material available at 10.1186/s13018-023-04315-1.

## Introduction

The posterolateral tibial plateau (PL) fractures are common in clinical practice, accounting for 15%–44.2% of all tibial plateau fractures [1–3]. Since the posterolateral tibial plateau fractures (PLFs) are located in the weight-bearing region of the tibial plateau as the tibia-femoral joint flexes [4, 5], open reduction and internal fixation is recommended for such fragments and any failure to stabilize it would result in knee instability [6]. Besides, because of the unique anatomy of posterolateral corner and complexities in exposure, reduction and fixation through the traditional anterolateral (AP) approach, the treatment of PLFs is a challenge for the orthopedic surgeons [7–10]. Until now, numerous techniques have been introduced in literature [11–17], however, the treatment for PLFs still remains controversial [18].

The rim plate fixation is an effective method to treat PLFs and has been accepted by a number of surgeons [1, 7]. The “Barrel Hoop Plate (BHP)” fixation which is an improvement of rim plates has achieved satisfactory preliminary clinical results [19]. Recently, a biomechanical study has compared the BHP with another two classical internal fixation patterns which were anterolateral plate (ALP) and posterolateral plate (PLP), and demonstrated that the BHP had a greater capacity to resist displacements along three-dimensional axes in PLFs [20]. However, according to previous morphological study, there are two main patterns of PLFs, PL-split and PL-depression [21]. Due to the difficulties in modeling establishment, only the Synbone models of PL-split were produced in this biomechanical study, and the PL-depression type, which is also a common subtype of PLFs and should be utilized to test the capacity of the fixations, has not been assessed.

**Fig. 1:**
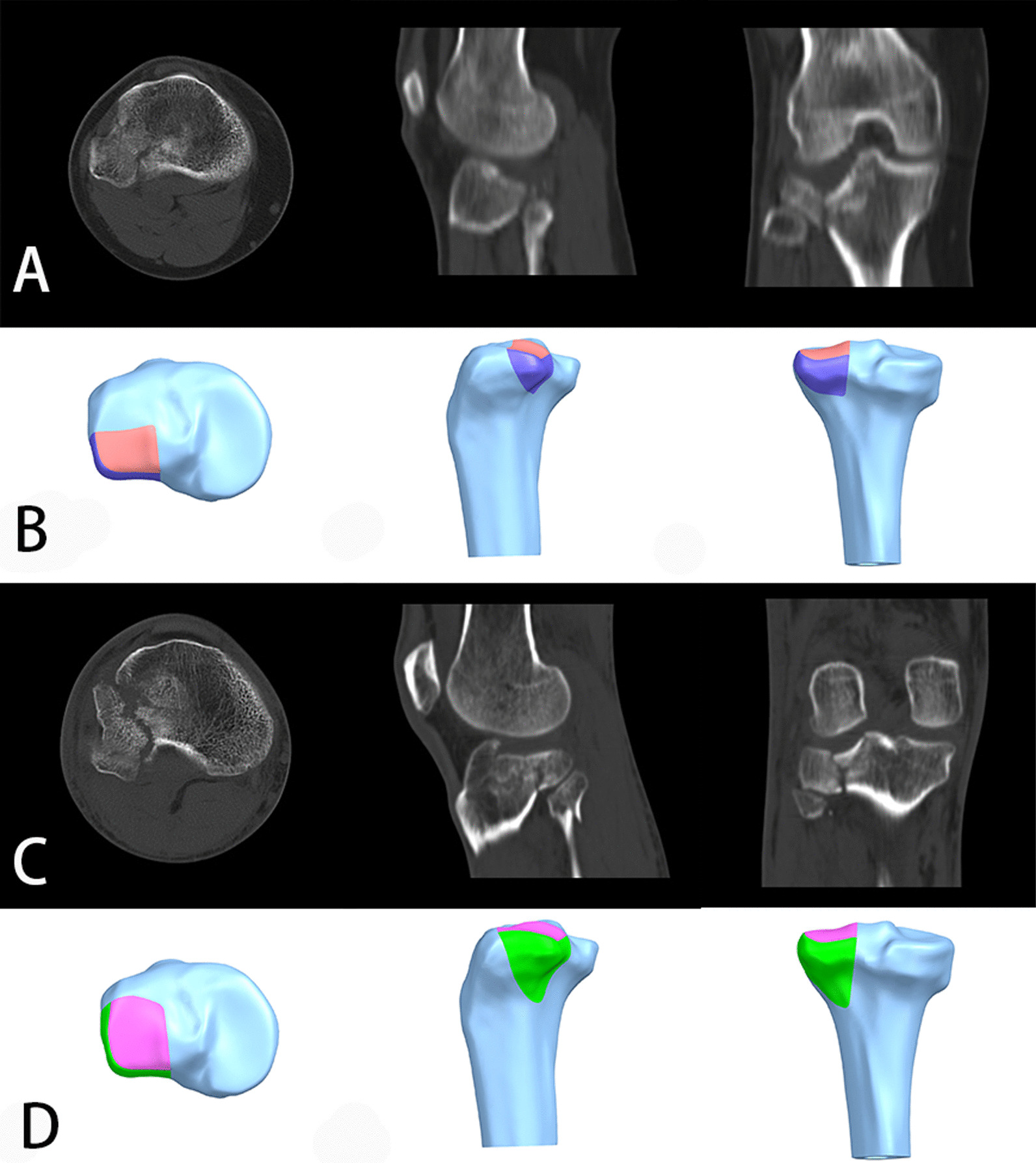
**A** the computed tomography images of a mild slope-type depression fracture (MSDF); **B** the modeling diagram of MSDF; **C** the computed tomography images of a local sink hole-type depression fracture (LSDF); **D** the modeling diagram of LSDF

Finite element analysis (FEA) is a widely-used method in the field of orthopedic research to analyze and predict the outcome of operative treatment, through converting three-dimensional models of bone-implant construct into finite elements with the application of simulated physiological loads [22]. Biomechanical studies via computational simulation can provide deeper insight into the stability and functionality of bone constructs, in specific, in the condition of various parameters, such as loading forces, fracture types and fixation patterns, which were difficult to simulate via in vitro artificial or cadaveric experiments [23–25]. In this study, two kinds of PL-depression models involving the mild slope-type depression fracture (MSDF) and the local sink hole-type depression fracture (LSDF) were both created (Fig. 1) [19], and the comprehensive biomechanical capacity of the BHP implant system and the other two conventional implant systems (ALP and PLP) were analyzed and compared by FEA method. We hope our study will provide useful biomechanical information about the fixations in the treatment of PL-depression tibial plateau fractures.

## Materials and methods

### Modeling of PL-depression PLFs and plate-screw systems

Computed tomography images (SOMATOM Definition AS1; Siemens, thickness, 0.6 mm; resolution, 512 × 512 pixels) in the Digital Imaging and Communications in Medicine (DICOM) format of the left lower limb of a healthy adult man, who was 33 years old, 178 cm in height, and 70 kg in body weight, were obtained and imported into the Mimics software (v20.0, The Materialise Group, Leuven, Belgium) to create a three-dimensional (3D) model. Then, the previously built model was imported to Geomagic Studio 2014 Software (3D system Inc., Rock Hill, SC, USA) for smoothing and polishing the surface.

**Fig. 2:**
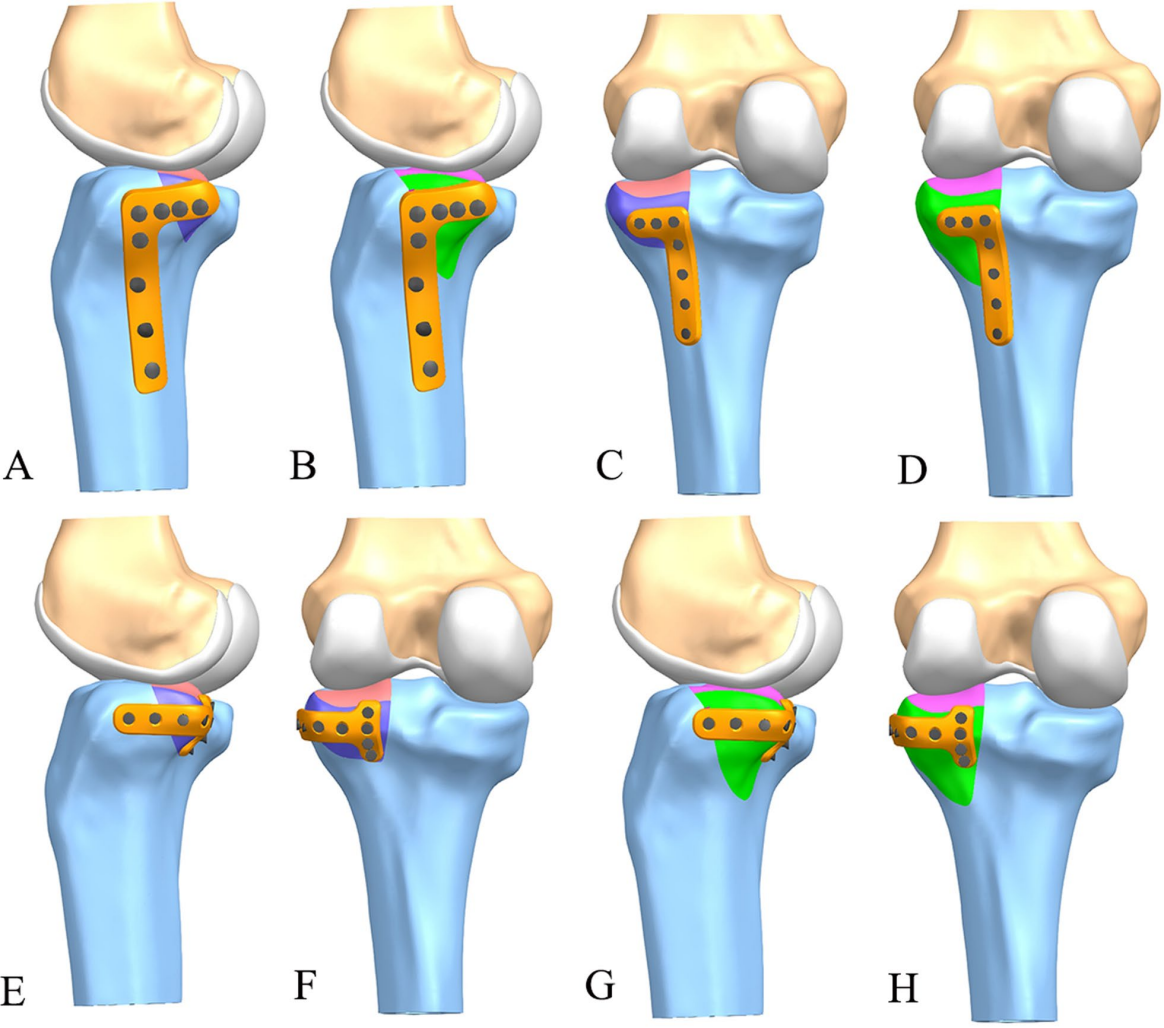
**A** the modeling diagram of ALP (a 3.5 mm proximal tibia locking compression plate) in MSDF; **B** the modeling diagram of ALP in LSDF; **C** the modeling diagram of PLP (tailored from a 2.7 mm distal radius locking plate) in MSDF; **D** the modeling diagram of PLP in LSDF; **E** and **F**, the modeling diagram of BHP (tailored from a 2.7 mm distal radius locking plate) in MSDF; **G** and **H**, the modeling diagram of BHP in LSDF

The 3D models of three kinds of plate-screw system were created according to the specifications of the manufacturer using Siemens NX 1911(Siemens Inc., Berlin, Germany). Group I was the ALP system, which was a 3.5 mm proximal tibia locking compression plate (Synthes GMBH, Oberdorf, Switzerland), comprising of eight screws, each with a diameter of 3.5 mm (four locking screws transversely placed proximally and four locking screws longitudinally placed distally). Group II was the PLP system, which was tailored from a 2.7 mm distal radius locking plate (Synthes GMBH, Oberdorf, Switzerland), comprising of seven screws, each with a diameter of 2.7 mm (three locking screws transversely placed proximally and four locking screws longitudinally placed distally). Group III was the BHP system, tailored from a 2.7 mm distal radius locking plate (Synthes GMBH, Oberdorf, Switzerland), which comprised nine screws, each with a diameter of 2.7 mm (four locking screws longitudinally placed medially and five locking screws placed transversely) (Fig. 2). All three plate types were similar to those in the study by Zhang et al. [20]

The 3D models of tibia were then imported into Siemens NX 1911(Siemens Inc., Berlin, Germany). The morphology of the two kinds of PL-depression models, the MSDF and the LSDF, were built according to previous studies [19, 21]. The plate-screw systems were then placed in the right place simulating fracture fixation models. There were six models for the fracture fixations: MSDF-ALP, MSDF-PLP, MSDF-BHP and LSDF-ALP, LSDF-PLP, LSDF-BHP (Fig. 2). Meshing and subsequent establishment of the finite element model were also performed with Ansys workbench 2019 (ANSYS Company, USA) (detailed in Additional files 1 and 2). A ten-node tetrahedron body element (C3D10) was used for the bone, plates, and screws, similar to previous studies [26]. The number of nodes and elements for all models were recorded (detailed in Additional file 3).

### Material properties

**Fig. 3:**
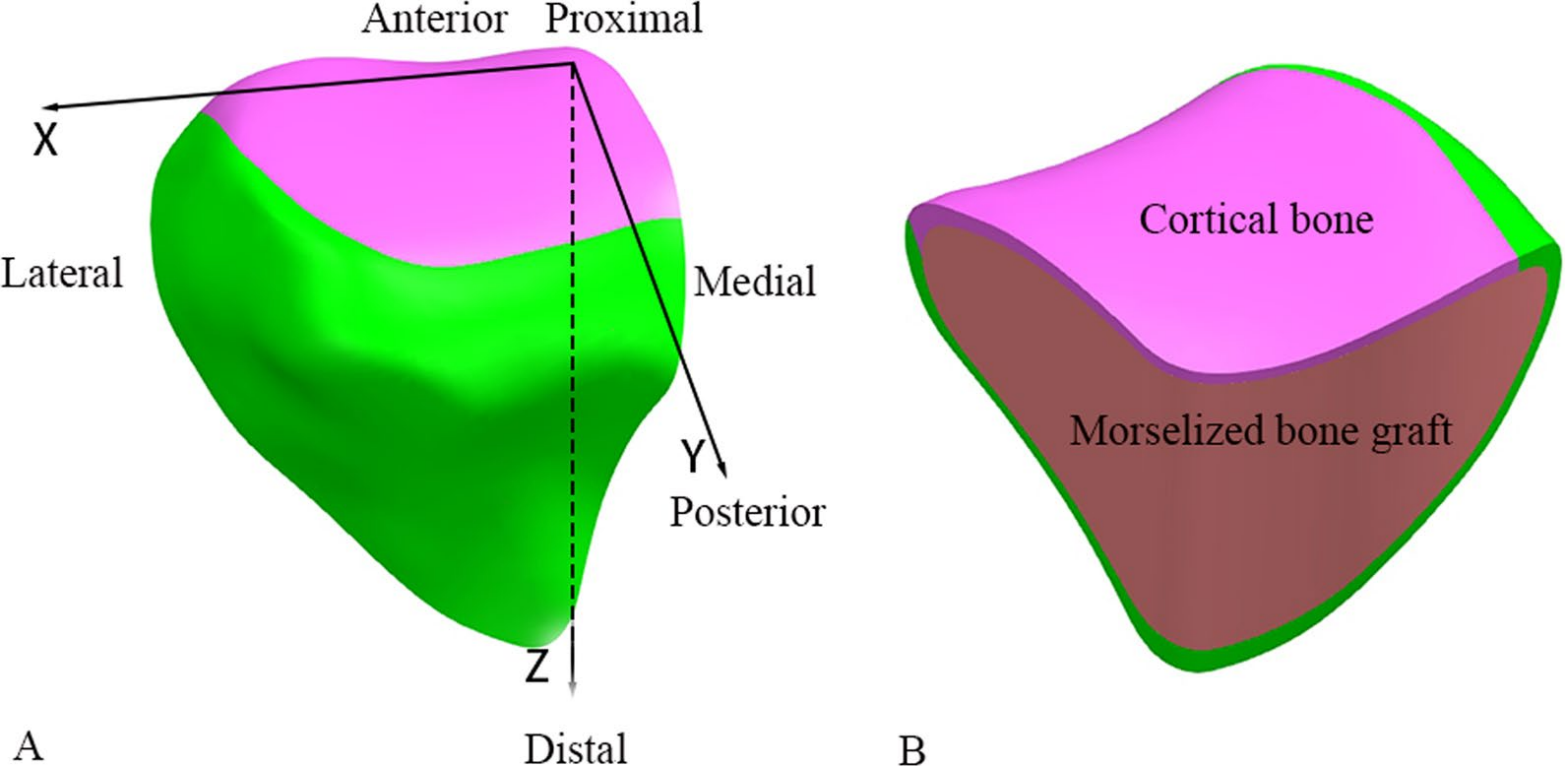
**A** the coordinate system: X, Y, and Z axes were defined as medial to lateral, anterior to posterior and proximal to distal, respectively; **B** the depression type PLFs were combined with a “cortical bone shell” and a “morselized bone graft core”

The material properties of the plate-screw system which were assumed to be homogeneous, isotropic, and linear elastic, were assigned as titanium alloy, with Young’s modulus (E) of 110 GPa and a Poisson ratio of 0.3 according to the product specifications [24, 27–30]. Young’s modulus of 17 GPa and 5GPa were set as a mean stiffness for the cortical bone and the cancellous bone, respectively. The modeled tibia consisted of cortical shell with a trabecular core, and the Poisson ratio was 0.3. In specific, before the final fixation, the PL-depression was routinely filled with morselized bone graft, as we applied in our clinical practice [19]. Therefore, Young’s modulus for the bone graft depression site was set at 150 MPa with Poisson ratio of 0.2, representing the high-strength bone graft values according to the literature [31] (Fig. 3).

### Loading and boundary conditions

Given most of the time, the knee was in an almost extended gesture, so the working condition of our FEA model was set in an extended knee [32]. The distal tibia was set to be immobile and the femur was kept in the same axis to simulate the loading condition of a knee joint. An axial force of 400N was applied from the distal femur to the tibial plateau [29, 33] (detailed in Additional file 4). The contact between screws and plate was assumed to be fully ensured and the interface was considered perfectly bounded [34]. A frictional contact with a coefficient of 0.4 was assumed to exist between the fragments [26]. Besides, the implants and bones were assumed as indirectly contact with a frictional coefficient of 0.3 [35].

### FEA stimulation and parameters for analysis

**Fig. 4:**
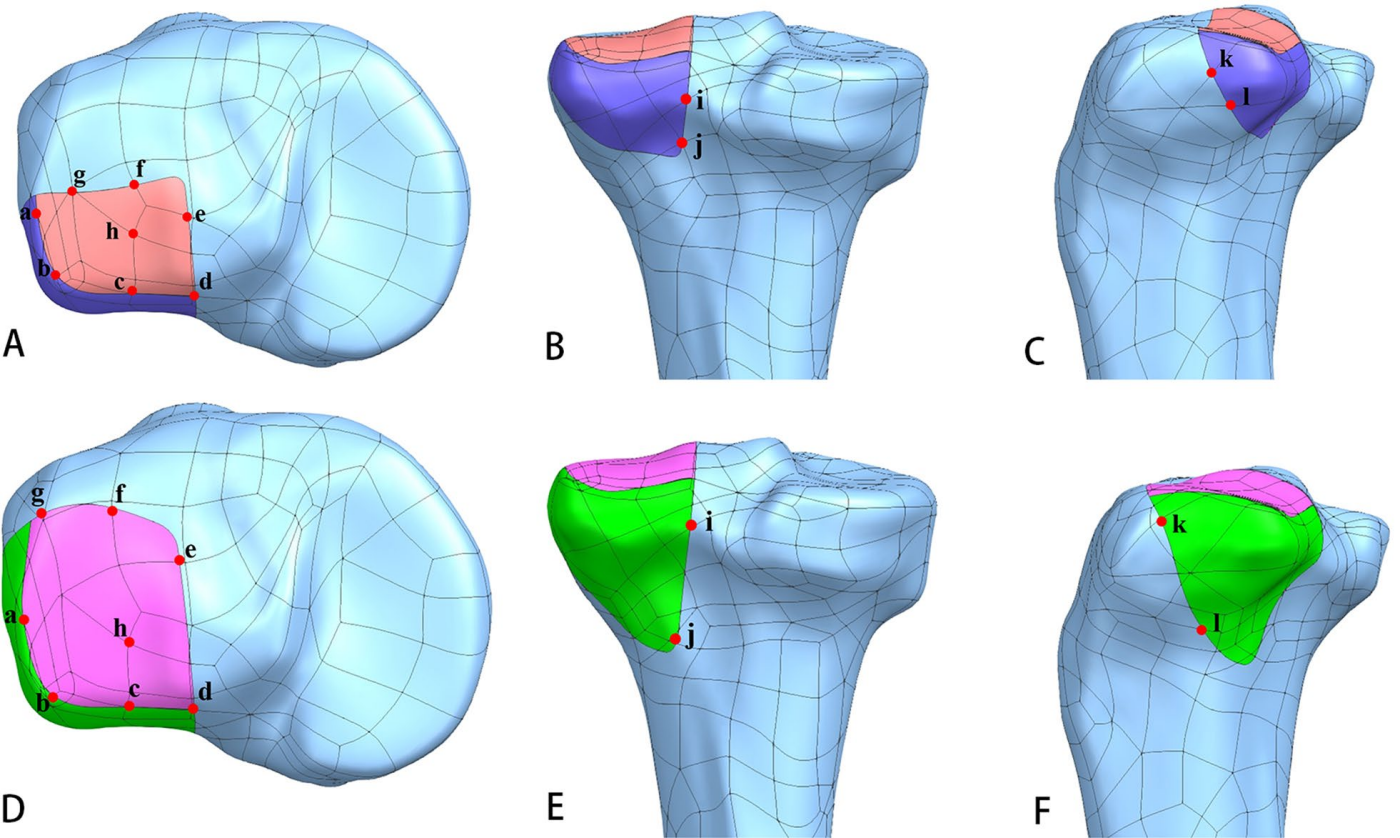
The key points of MSDF and LSDF. **A** and **D**: a, b, c and d were the points distributed in the rim of the depression, e, f and g were distributed in the fracture line of the articular surface, and h was in the center of the depression; **B** and **E**: i and j were distributed in the fracture line of the posterior wall; **C** and **F**: k and l were distributed in the fracture line of the lateral wall

The finite element models were analyzed by Optistruct software (v13.0, Altair Engineering Inc., Troy, MI, USA). Prior to FEA analysis, a coordinate system was created where the positive directions of X, Y, and Z axes were defined as medial to lateral, anterior to posterior and proximal to distal (Fig. 3A). In our study, the displacements of PLFs and stresses of the different fixations were output. In detail, the maximal displacements of the PLFs in three axes were recorded and the hot spot map of the displacements were delineated as well. The key points (mainly distributed in the fracture lines and articular surface) of the two fracture models were punctuated (Fig. 4), and the displacements of same points were compared between each fixation group. To be specific, the Point a, b, c and d which were distributed in the rim of the depression were set to test the stability of the rim after fixation, while the Point e, f and g which represent the fracture line boundary of the articular surface after fixation (Fig. 4A, D). Point h was in the center of the depression (Fig. 4A, D). Point i and j were distributed in the fracture line of the posterior wall (Fig. 4B, E). Point k and l were in the fracture line of the lateral wall (Fig. 4C, F). The maximal Equivalent (von-Mises) Stress of the three fixation systems, and the maximal shear stress of the PLFs were summarized. Besides, the stiffness of the fixation was calculated.

## Results

### The displacement of PLFs

#### The maximal displacements

**Fig. 5:**
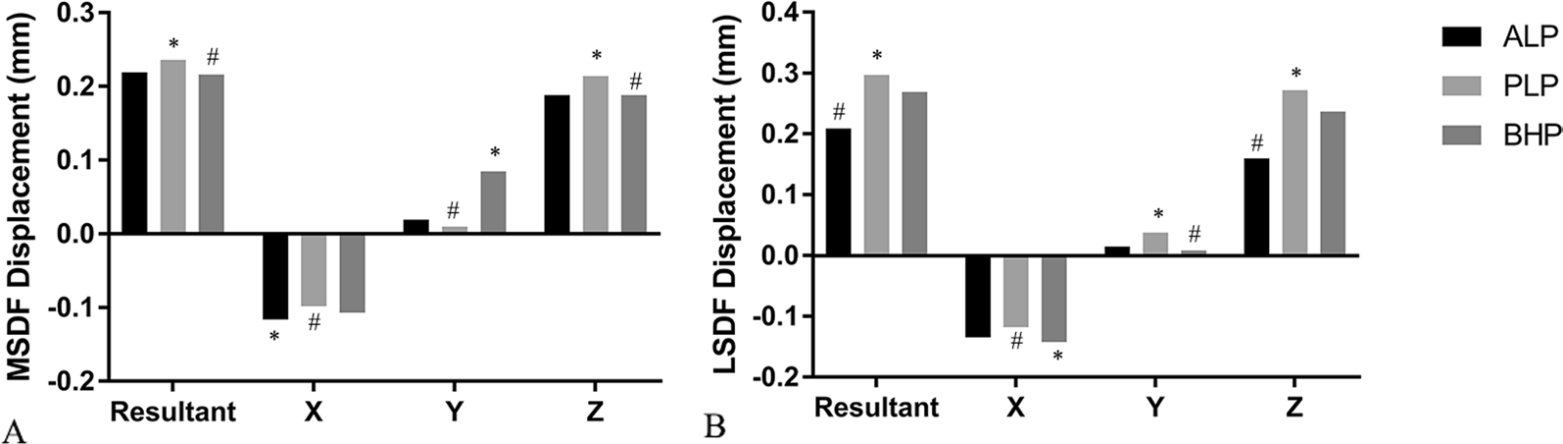
**A** the resultant and component maximal displacements of MSDF; **B** the resultant and component maximal displacements of LSDF. X: the X axis (medial to lateral); Y: the Y axis (anterior to posterior); Z: the Z axis (proximal to distal). *: the maximum value; #: the minimum value

The maximal displacements of all the PLFs including resultant and component in three axes are listed in Fig. 5. As it demonstrates, the main deformation occurred along the Z axis, while the deformation in X axis and Y axis were much less. The maximal displacement of MSDF was least in Group BHP (0.216 mm), which was followed closely by the Group ALP (0.219 mm) and then the Group PLP (0.236 mm). While the maximal displacement of LSDF in Group ALP (0.209 mm) was the least and about 30% less than that in Group PLP (0.297 mm) and 22% less than that in Group BHP (0.269 mm). As the displacement was divided into the three axes components, the similar tendency was maintained regarding the Z axis: MSDF-BHP (0.188 mm) = ALP (0.188 mm) < PLP (0.214 mm), LSDF-ALP (0.160 mm) < BHP (0.237 mm) < PLP (0.272 mm) (Fig. 5). Besides, compared with the maximal displacements of MSDF, Group PLP and BHP of LSDF were larger, however, the displacements in Group ALP was less in Model of LSDF.

#### The distribution of the articular surface displacements

**Fig. 6:**
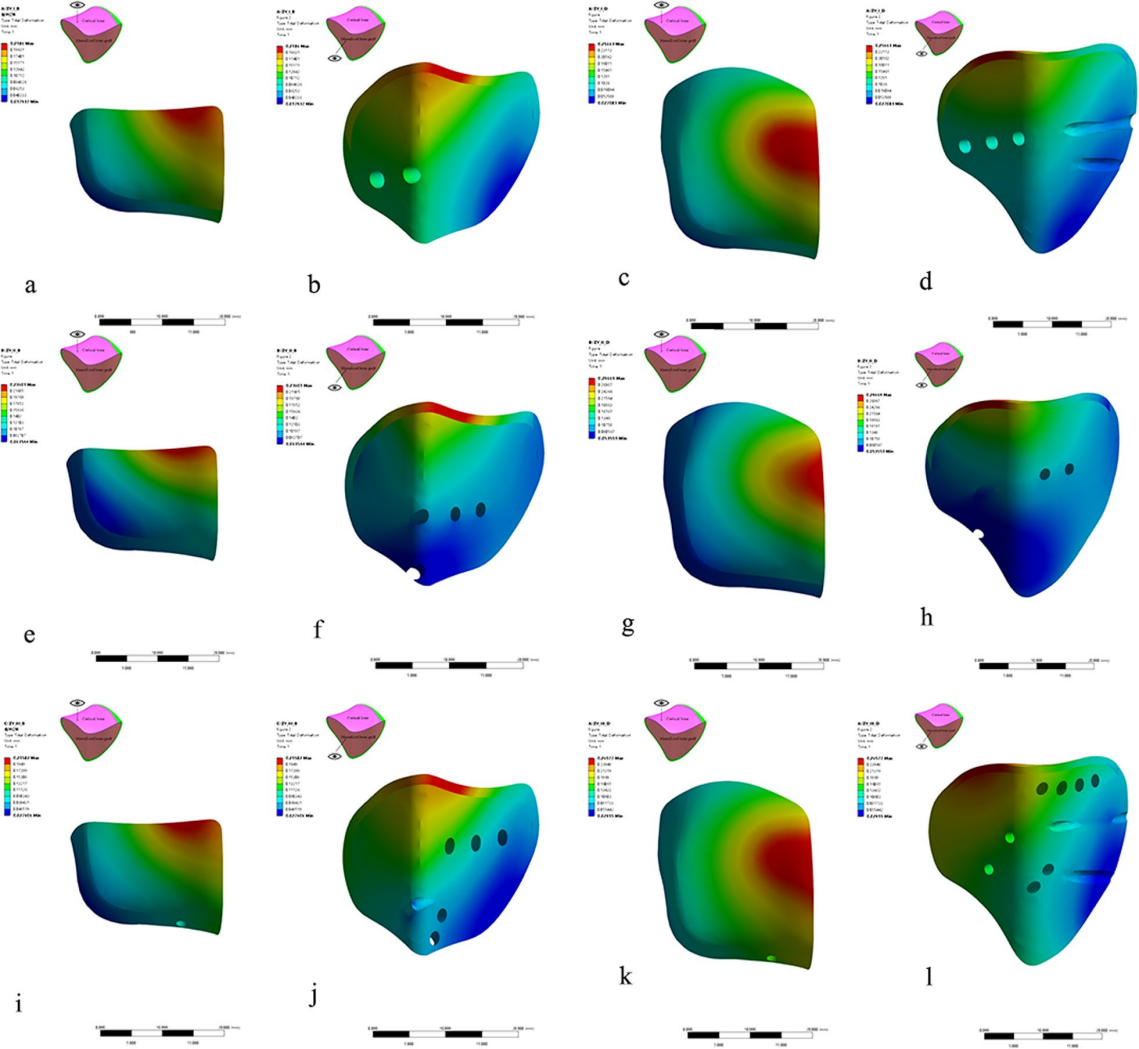
The distribution of total deformation of all the FE models. **a** and **b**: the deformation map of ALP-MSDF; **c** and **d**: the deformation map of ALP-LSDF; **e** and **f**: the deformation map of PLP-MSDF; **g** and **h**: the deformation map of PLP-LSDF; **i** and **j**: the deformation map of BHP-MSDF; **k** and **l**: the deformation map of BHP-LSDF. The figures of **a**, **e**, **i**, **c**, **g**, **k** are observed from the top to the fragment, while the figures of **b**, **f**, **j**, **d**, **h**, **l** are observed from the inside of the fragment

The distribution of total deformation of all the FE models is shown in Fig. 6. The maximal deformation region was the anteromedial articular surface of MSDF and the central medial articular surface of LSDF. The displacements decreased in a wavy shape from the center of the maximal regions to the periphery.

#### The displacements of the key points

**Fig. 7:**
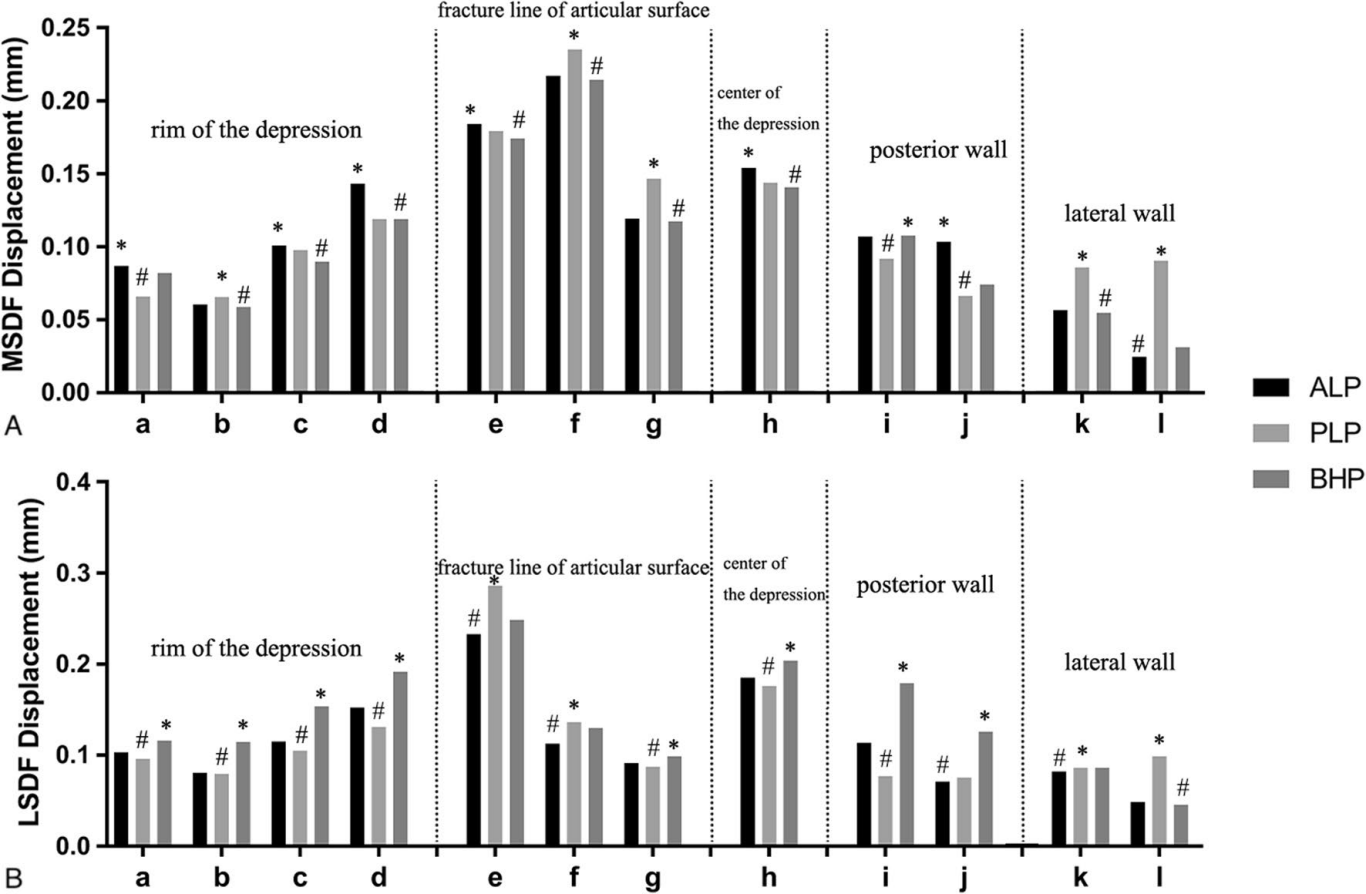
**A** the displacements of the key points in MSDF. **B** the displacements of the key points in LSDF. a, b, c and d: the points in the rim of the depression; e, f and g: the points in the fracture line of articular surface; h: the point in the center of the depression; i and j: the points in the posterior wall; k and l: the points in the lateral wall. *: the maximum value; #: the minimum value

In the model MSDF, the displacements of Points a (0.0821 mm), b (0.0587 mm), and c (0.0898 mm) in Group BHP were all mild (Fig. 7A-a, b, c). The displacement of Point d showed a high value of 0.143 mm in Group ALP, which was about 21% more than the other two fixation patterns (Fig. 7A-d). The displacement of point e of the three internal fixation patterns was close, around 0.18 mm (Fig. 7A-e). The displacements of PLP at Points f (0.235 mm) and g (0.147 mm) were about 10% and 25% more than the other two groups, respectively (Fig. 7A-f, g). The displacement of the Point h was least in BHP (Fig. 7A-h). For Point i, j, the displacements of the PLP were minimal. The displacement of ALP at Point j (0.103 mm) was the largest, which was 56% larger than that of PLP (0.0663 mm) and 39% larger than that of BHP (0.0741 mm) (Fig. 7A-I, j). At Point k, l, the displacement of PLP was higher than the other two fixation methods (Fig. 7A-k, l).

In the model LSDF, the displacements of Points a, b, c, d were least in Group PLP (0.0959 mm, 0.0794 mm, 0.105 mm, 0.131 mm), and were most in Group BHP (0.116 mm, 0.115 mm, 0.154 mm, 0.192 mm) (Fig. 7B-a, b, c, d). The displacements of the PLP at points e, f and g were more than those of the other two fixation methods (Fig. 7B-e, f, g). At the Point h of the articular surface, the displacement of BHP (0.203 mm) was about 11% more than that of the other two groups (Fig. 7B-h). Point i, j, also showed the smallest displacement in PLP and the largest in BHP (Fig. 7B-I, j). The displacements of point k were close to each other, and the displacement PLP of point l is the largest (0.098 mm), which was 110% more than the other two fixation methods (Fig. 7B-k, l).

### Stresses in the fixation complex

#### The maximal equivalent (von-Mises) stress of implants

**Fig. 8:**
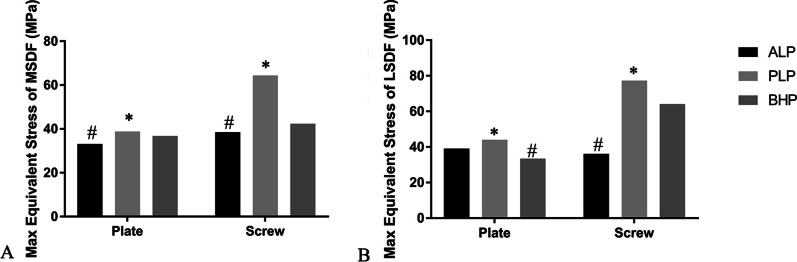
**A** the maximal Equivalent (von-Mises) Stress of the plates and screws in MSDF; **B** the maximal Equivalent (von-Mises) Stress of the plates and screws in LSDF. *: the maximum value; #: the minimum value

The maximal Equivalent (von-Mises) Stress of the three fixation systems in two kinds of PLFs is summarized in Fig. 8. PLP system demonstrated the most maximal Equivalent (von-Mises) stress among three fixation patterns, both on plates and screws. The according values of the stress of the plate and screws in MSDF were 38.685 MPa and 64.399 MPa, respectively. And in the LSDF group, the max Equivalent Stress was 44.017 MPa on plate and 77.265 MPa on screws.

**Fig. 9:**
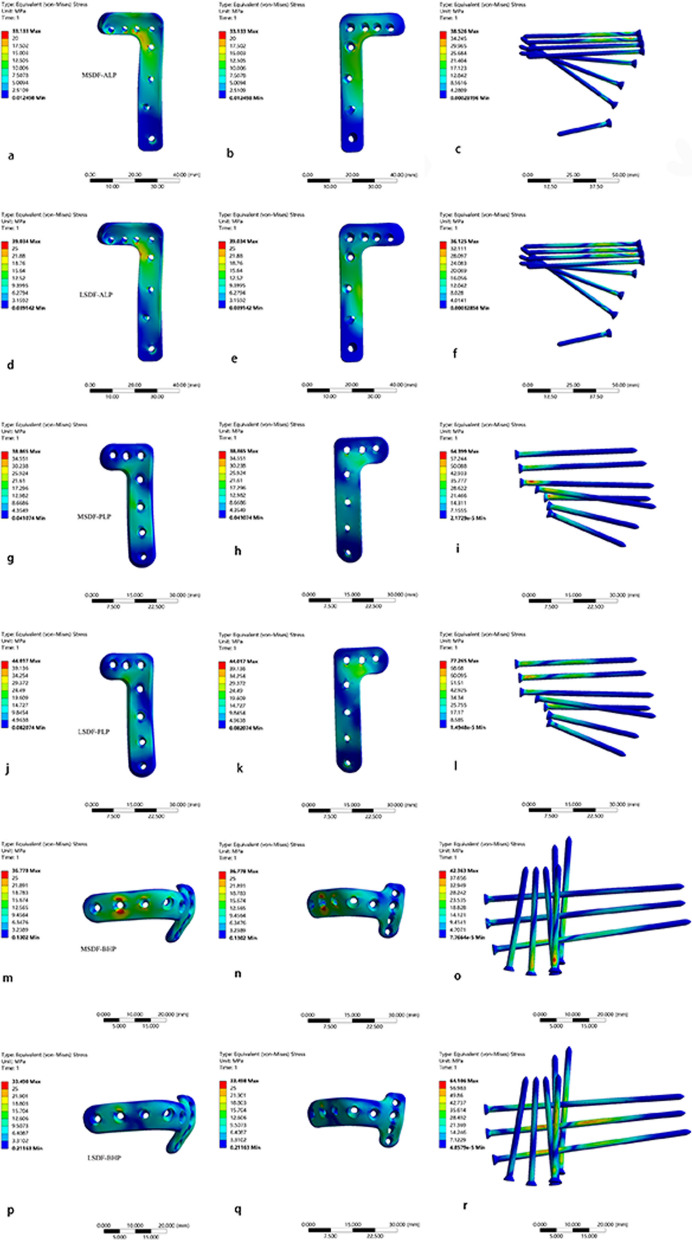
The stress map of internal fixation systems. **a**, **b**, and **c**: the stress map of ALP-MSDF; **d**, **e** and **f**: the stress map of ALP-LSDF; **g**, **h** and **i**: the stress map of PLP-MSDF; **j**, **k** and **l**: the stress map of PLP-LSDF; **m**, **n** and **o**: the stress map of BHP-MSDF; **p**, **q** and **r**: the stress map of BHP-LSDF

Additionally, the stress map is presented in Fig. 9. The Equivalent stresses of the ALP in MSDF and LSDF were both concentrated around the turning point of the inverted L shape (Fig. 9a, b, d, e). The Equivalent stresses of the PLP in the two PLFs were concentrated around the first two screw holes in the vertical part of the plates (Fig. 9g, h, j, k). The Equivalent stresses of the BHP were relatively evenly distributed in MSDF and LSDF. The only stress concentration area was located around the second screw hole of the transverse arm (Fig. 9m, n, p, q).

#### The max shear stress of PLFs

**Fig. 10:**
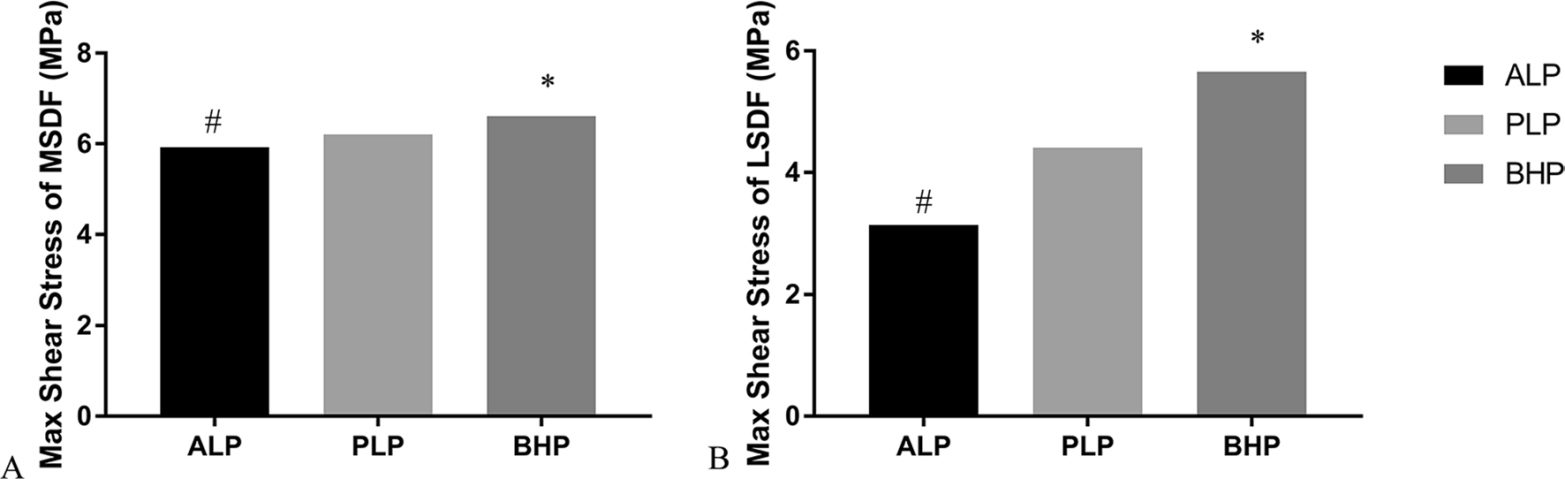
**A** The max shear stress of MSDF; **B** the max shear stress of LSDF. *: the maximum value; #: the minimum value

Among all three fixation patterns, the max shear stress of MSDF and LSDF were lowest (5.921 MPa, 3,136 MPa) in ALP system and highest in BHP system (6.608 MPa, 5.658 MPa) (Fig. 10).

### Stiffness of the fixation

**Fig. 11:**
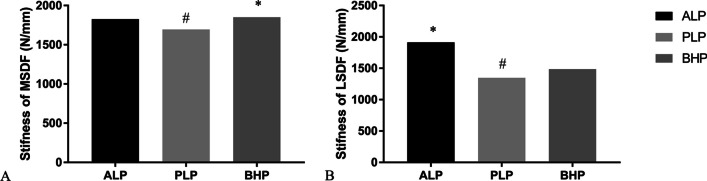
**A** the stiffness of all the three fixation systems in MSDF; **B** the stiffness of all the three fixation systems in LSDF. *: the maximum value; #: the minimum value

The stiffness of all the three fixation systems in two kinds of PLFs is shown in Fig. 11. ALP and BHP showed a comparable stiffness in MSDF and ALP had the strongest stiffness in the fixation of LSDF.

## Discussion

In general, open reduction and internal fixation are the fundamental ways for the treatment of the PL-depression tibial plateau fractures, so it is crucial to effectively restore the anatomy of the PLFs during the operation. If the reduction and fixation of the PLFs are not done appropriately, the PL articular surface collapse may affect the knee stability and a salvage surgery would be inevitable [6]. Numerous strategies have been proposed to deal with this intractable problem [1, 11–17, 36], and recently, a BHP system for fixing the PLFs has achieved acceptable preliminary clinical results [19, 20]. The associated biomechanical results also demonstrated a similar advantage for the fixation of the PL-split fractures [19, 20]. However, a limitation of the study was the setup of the model, which represented only one type of the PLFs, lacking the evaluation of the depression scenario. Furthermore, based on the biomechanical studies from the database, most of which were just focused on the PL-split, none of them explored the fixation effect of the PL-depression type [20, 37, 38]. To the best of our knowledge, the biomechanical characters were quite different between depression and split, and mean while, the depression pattern is essential for testing the stability of the implant-model complex. Given that the uniform depression type could not be mimicked by routine biomechanical modeling methods, our study is the first to build depression models and test them by means of FEA [24, 26, 28].

After FEA simulation, the maximal displacement showed different results among all three fixations in the treatment of the depression types. In MSDF, the maximal displacement was minimal in BHP group, indicating that BHP was most suitable for the MSDF type depression. The PLP was not appropriate for this depression pattern as it had the most displacement. After the decomposition of the resultant displacement, *Z*-axis sustained the most weight. Both BHP and ALP groups showed the minimal *Z*-axis displacements, while PLP had the maximal value among the three fixations. In LSDF, however, BHP does not demonstrate a similar biomechanical stability as in MSDF, and it is the ALP to have a stronger capacity than the other two implants, both in resultant and *Z*-axis components. Likewise, PLP acted disappointingly in LSDF group. Compared with the maximal displacements of MSDF, the displacements in Group ALP were less in Model of LSDF which was opposite to the other two fixation methods. This may be because LSDF was held by more screws than MSDF in the ALP system. As a result, ALP possess a general better capability in the treatment of the PL-depression, and BHP could only be selected to fix MSDF, instead of LSDF. PLP is not recommended for PL-depression based on our current FEA study.

Besides the maximal displacement, key points displacements around the depression should also be analyzed and compared. It can be found as the BHP was applied in MSDF, displacements of the PL rim (Points a, b, c), depression center (Point h) were the least among three fixation types, however, the most in LSDF. It can be interpreted that as MSDF was fixed by the BHP, the barrel hoop could effectively fix the fragments transversely of both the fragment and the normal proximal tibia. As the size of the bone fragment increased from MSDF to  LSDF, the barrel hoop screws were mainly fixed in the fragment, without holding enough normal bone, which might decrease the final stability. Therefore, the “barrel hoop” effect of the rim is reduced as the depression area increased because it needs more healthy bone to support. However, PLP showed the best PL rim fixation effect because this fixation pattern is most consistent with the injury mechanisms. Point D was a relatively special point, which was considered as a “blind zone” where the surgeon is hard to visualize from the conventional AL approach. It is difficult to fix effectively by neither ALP nor BHP, but  PLP showed a better ability to hold this region by the least displacement after loading. At the Point g and f in MSDF and LSDF, the fixation effect of PLP was poor. It was probably because the fracture line was far away from the PLP, decreasing the purchase ability. Conversely, PLP worked best at Points i and j, because it directly supported at these two positions. In a similar way, ALP maintained the lateral Points k and l stronger than the other two fixation methods. This phenomenon could also be interpreted by that the buttress fixation based on the injury force mechanisms provides the best local point stability.

In terms of stress distribution, it can help us to better understand the different working mechanisms of different internal fixation methods in the fixation of PLFs. The stress of ALP was concentrated at the corner of inverted L-shape, because the L-shaped transverse screw was connected to the PLF, while the vertical screws were mainly connected to the tibia shaft. When the loading was applied, the two kinds of screws closed to the fracture line rotated and displaced at this turning point, so the stress was concentrated here. The stress of PLP was concentrated in the middle and upper segment of the vertical arm, where was the boundary between the lower tip of PLFs and the normal tibia shaft. The "barrel hoop effect" effectively dispersed the stress, so that the stress of the BHP could be distributed evenly in both MSDF and LSDF. For the stress of bones, the max-shear stress was calculated to show the risk of trabecular microfracture which might bring the screw loosening, leading to the failure of ORIF [26]. Among all three fixations, ALP showed the minimal shearing stress and the BHP showed the maximal, which was considered to be the implant morphology related. The transverse implant pattern should have the maximal shearing stress.

There are several limitations in our study which need to be elucidated. First of all, the proximal tibia-fibular articulation has not been well defined as the proximal fibular may play a role in the weight bearing after the fixation. Future studies need to be designed to illustrate this question. Secondly, although the bearing of the PL part of the tibial plateau only occurs in knee extremely flexes, which only covers a little proportion in daily life, it still needs to be mimicked in future FEA study. Thirdly, the uniformed cortical and cancellous bone were set up in the current FEA modeling, instead of the trabecular bone morphology and construction stimulation, which also should be more precise in future study design.

## Conclusion

In the mild slope-type depression fracture (MSDF), the BHP has the best biomechanical capacity, especially in displacements of key points such as the PL rim, fracture line, and depression center. In the local sink hole-type depression fracture (LSDF), the ALP shows the best biomechanical effect. Although the PLP has the best fixation effect on the posterior wall, it is not suitable for PL-depression fixation.

## Funding

This study was supported by the Youth Program of National Natural Science Foundation of China (No. 82002287) and Clinical Research Fund of Shanghai Sixth People’s Hospital (ynlc201901).

### Supplementary Information

**Additional file 1: Fig. S1**. The mesh structure in the local sink hole-type depression fracture (LSDF). A and B, ALP (a 3.5mm proximal tibia locking compression plate); C and D, PLP (tailored from a 2.7 mm distal radius locking plate); E and F, BHP (tailored from a 2.7 mm distal radius locking plate).

**Additional file 2: Fig. S2**. The mesh structure in the mild slope-type depression fracture (MSDF). A and B, ALP (a 3.5mm proximal tibia locking compression plate); C and D, PLP (tailored from a 2.7 mm distal radius locking plate); E and F, BHP (tailored from a 2.7 mm distal radius locking plate).

**Additional file 3: Fig. S3**. A and B, the loading structure of the biomechanical system.

**Additional file 4: Table S1**. The number of elements and nodes of the models
