# Supplementary figures and images for: Biomechanical study of a new rim plate fixation strategy for two kinds of posterolateral depression patterns of tibial plateau fractures: a finite element analysis
Source: J Orthop Surg Res. 2023 Nov 7;18:840. doi: 10.1186/s13018-023-04315-1 (PMC10629018; doi:10.1186/s13018-023-04315-1)

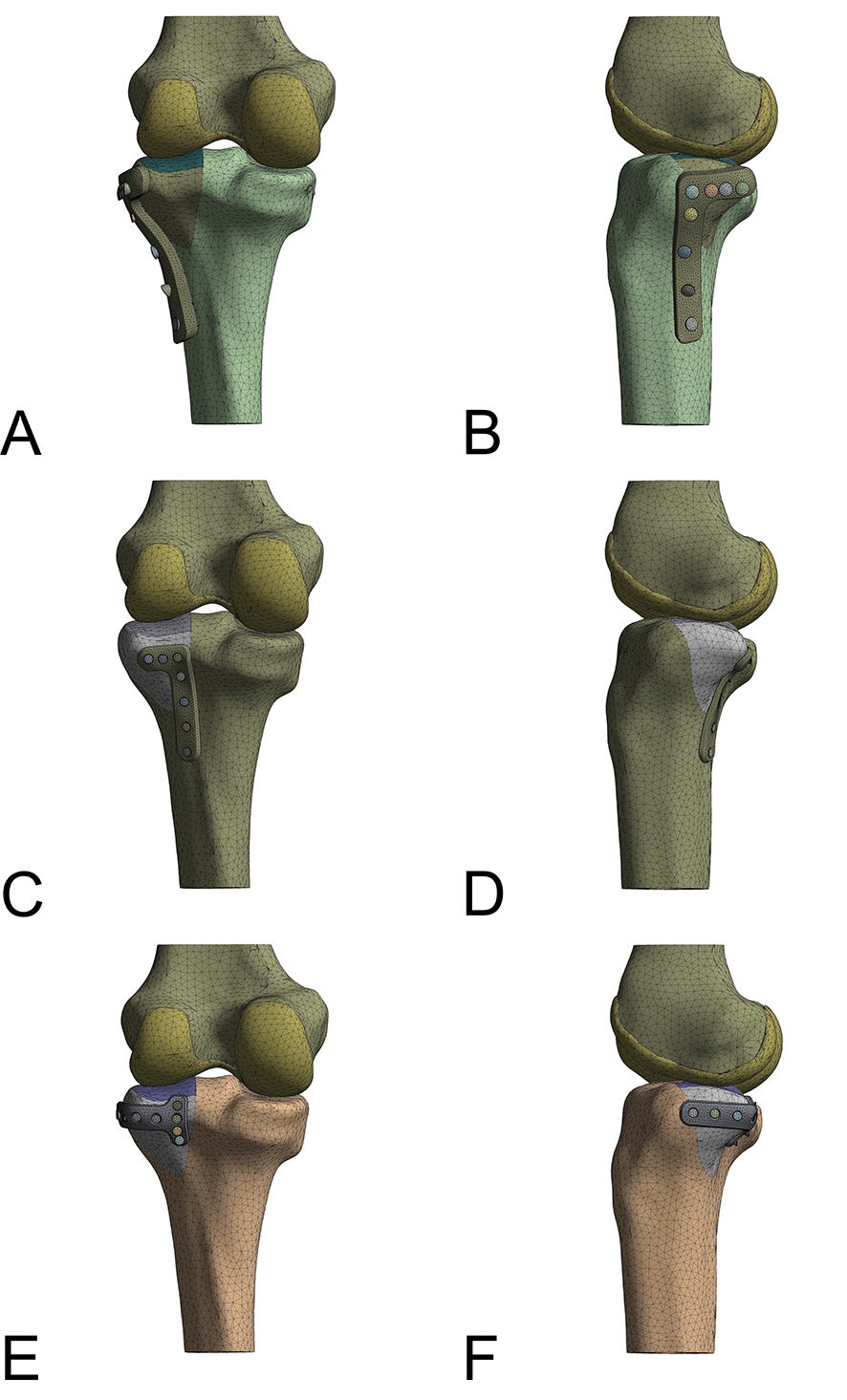

Supplement: Supplementary file 1 — Additional file 1: Fig. S1. The mesh structure in the local sink hole-type depression fracture (LSDF). A and B, ALP (a 3.5mm proximal tibia locking compression plate); C and D, PLP (tailored from a 2.7 mm distal radius locking plate); E and F, BHP (tailored from a 2.7 mm distal radius locking plate). [file 13018_2023_4315_MOESM1_ESM.tif]

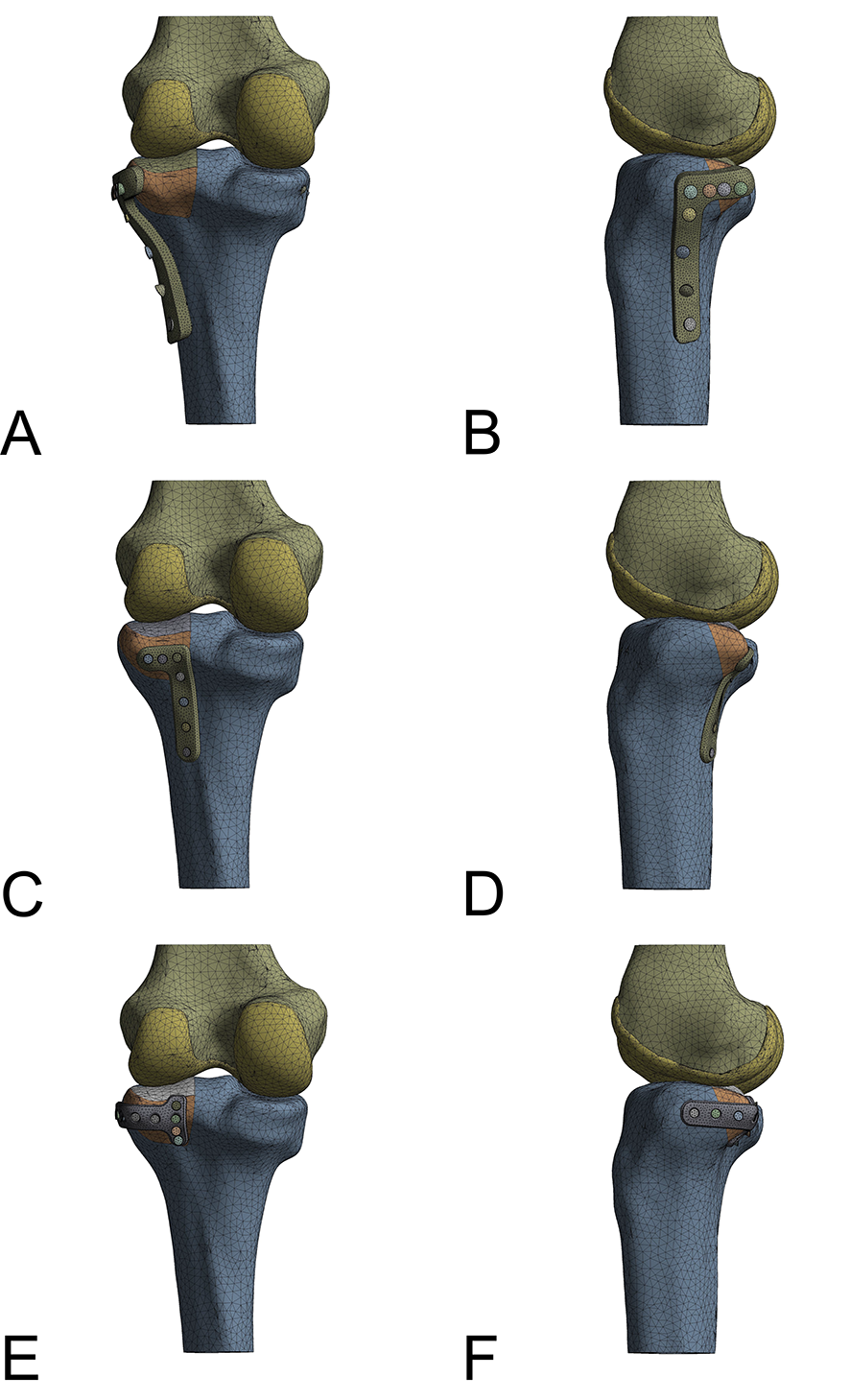

Supplement: Supplementary file 2 — Additional file 2: Fig. S2. The mesh structure in the mild slope-type depression fracture (MSDF). A and B, ALP (a 3.5mm proximal tibia locking compression plate); C and D, PLP (tailored from a 2.7 mm distal radius locking plate); E and F, BHP (tailored from a 2.7 mm distal radius locking plate). [file 13018_2023_4315_MOESM2_ESM.tif]

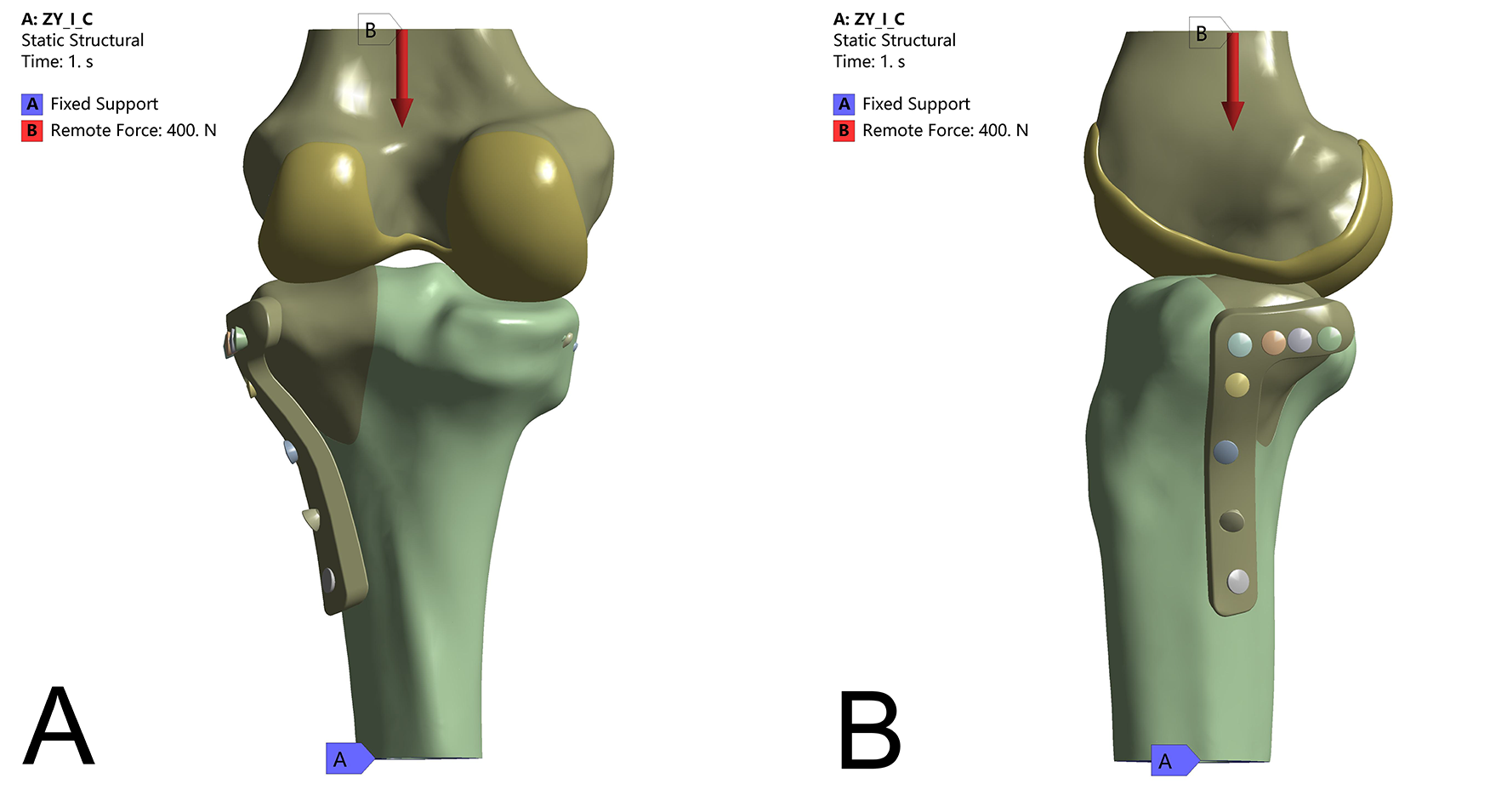

Supplement: Supplementary file 3 — Additional file 3: Fig. S3. A and B, the loading structure of the biomechanical system. [file 13018_2023_4315_MOESM3_ESM.tif]
