# Supplementary material for: Biomechanical study of a new rim plate fixation strategy for two kinds of posterolateral depression patterns of tibial plateau fractures: a finite element analysis
Source: J Orthop Surg Res. 2023 Nov 7;18:840. doi: 10.1186/s13018-023-04315-1 (PMC10629018; doi:10.1186/s13018-023-04315-1)
Supplement: Supplementary file 4 — Additional file 4: Table S1. The number of elements and nodes of the models [file 13018_2023_4315_MOESM4_ESM.docx]

**Table S1. The number of elements and nodes of the models**

| **Nodes/Elements** | MSDF | | | LSDF | | |
| --- | --- | --- | --- | --- | --- | --- |
|  | ALP | PLP | BHP | ALP | PLP | BHP |
| **Plates** | 56199/34938 | 33066/19979 | 39391/23712 | 56199/34938 | 33066/19979 | 39391/23712 |
| **Screws** | 69298/39773 | 39652/22074 | 56496/31485 | 69398/39773 | 39652/22074 | 56496/31485 |
| **Fragment-****cancellous bone** | 5196/2975 | 9916/6078 | 9237/5470 | 10127/6124 | 10832/6704 | 18674/11459 |
| **Fragment-cortical bone** | 3255/1648 | 4258/2196 | 5796/2997 | 4921/2465 | 5172/2617 | 7937/4170 |
| **Tibia-cancellous bone** | 52979/34755 | 53079/35217 | 68395/45604 | 50184/32861 | 48953/32361 | 64177/42879 |
| **Tibia-cortical bone** | 42414/23457 | 44404/24952 | 36395/20081 | 40269/22338 | 42707/24022 | 35663/19849 |
| **Total** | 229341/137546 | 184375/110496 | 215710/129349 | 231098/138499 | 180382/107757 | 222338/133554 |

ALP, anterolateral plate; PLP, posterolateral plate; BHP, barrel hoop plate; MSDF, mild slope-type depression fracture; LSDF, local sinkhole-type depression fracture.
